# Mammalian antiviral systems directed by small RNA

**DOI:** 10.1371/journal.ppat.1010091

**Published:** 2021-12-16

**Authors:** Tomoko Takahashi, Steven M. Heaton, Nicholas F. Parrish

**Affiliations:** 1 Department of Biochemistry and Molecular Biology, Graduate School of Science and Engineering, Saitama University, Saitama, Japan; 2 Genome Immunobiology RIKEN Hakubi Research Team, Cluster for Pioneering Research, RIKEN, Yokohama, Japan; 3 Center for Integrative Medical Sciences, RIKEN, Yokohama, Japan; Yale University School of Medicine, UNITED STATES

## Abstract

There are strong incentives for human populations to develop antiviral systems. Similarly, genomes that encode antiviral systems have had strong selective advantages. Protein-guided immune systems, which have been well studied in mammals, are necessary for survival in our virus-laden environments. Small RNA–directed antiviral immune systems suppress invasion of cells by non-self genetic material via complementary base pairing with target sequences. These RNA silencing-dependent systems operate in diverse organisms. In mammals, there is strong evidence that microRNAs (miRNAs) regulate endogenous genes important for antiviral immunity, and emerging evidence that virus-derived nucleic acids can be directly targeted by small interfering RNAs (siRNAs), PIWI-interacting RNAs (piRNAs), and transfer RNAs (tRNAs) for protection in some contexts. In this review, we summarize current knowledge of the antiviral functions of each of these small RNA types and consider their conceptual and mechanistic overlap with innate and adaptive protein-guided immunity, including mammalian antiviral cytokines, as well as the prokaryotic RNA-guided immune system, CRISPR. In light of recent successes in delivery of RNA for antiviral purposes, most notably for vaccination, we discuss the potential for development of small noncoding RNA–directed antiviral therapeutics and prophylactics.

## Author summary

Viruses are all around us and are likely inside some of the reader’s cells at this moment. Organisms are accommodated to this reality and encode various immune systems to limit virus replication. In mammals, the best studied immune systems are directed by proteins that specifically recognize viruses. These include diverse antibodies and T cell receptors, which recognize viral proteins, and pattern recognition receptors, some of which can recognize viral nucleic acids. In other organisms, including bacteria, immune systems directed by small RNAs are also well known; spacer-derived guide RNAs in CRISPR/Cas immune systems are one prominent example. The small RNAs directing these systems derive their specificity via complementary base pairing with their targets, which include both host and viral nucleic acids. Rather than having “traded in” these systems for more advanced protein-directed systems, increasing evidence supports the perspective that small RNA–directed immune systems remain active in mammalian antiviral immunity in some contexts. Here, we review what is known so far about the emerging roles of mammalian siRNAs, miRNAs, piRNAs, and tRNAs in directing immunity to viruses.

## Introduction

Viruses are the most abundant and genetically diverse entity in our biosphere and exist wherever life is found [1]. Viruses are characterized by dependence on host genome-encoded protein products and other materials (e.g., plasma membrane) for their replication, whereas host cellular and organismal replication are in principle independent of the genetic material of viruses. Replication of some viruses is associated with cellular and organismal death. Virus replication has thus often been interpreted to be detrimental to their hosts at evolutionary, organismal, and cellular scales of analysis (although host-virus antagonism is not a comprehensive picture of virus–host ecology, e.g., see [2]). Numerous systems that confer resistance to or otherwise mitigate virus infection have been identified. Immune systems capable of distinguishing “self” from “non-self” and eliminating “non-self” are inferred to have been established and refined through antagonistic interactions between viruses and their hosts over long periods of time [3–5]; mammalian genomes encode various complex systems known to mediate antiviral functions.

Components of mammalian immune systems are often categorized as belonging to innate or adaptive arms based on generalizably distinct functional and evolutionary features. When a virus infects human cells, innate immunity is activated first, followed by adaptive immunity. Innate immunity is activated by features common to invading pathogens, termed pathogen-associated molecular patterns (PAMPs). PAMPs are recognized by sensor proteins such as Toll-like receptors (TLRs) and RIG-I like receptors (RLRs) [6–9]. Upon activation, these receptors induce production of various antiviral cytokines, such as type-I interferon (IFN). Cytokines stimulate expression of antiviral genes, which repress viral replication and recruit or activate specialized cells including phagocytes and antigen-presenting cells. Adaptive immune lymphocytes generate vastly diverse receptors through gene recombination and then selectively proliferate on the basis of their receptor’s specificity. This enables high specificity as well as anamnestic properties dependent on long-lived “memory” cells. Conventional mammalian adaptive immunity is targeted to presented protein antigens and is characterized by relative evolutionary novelty; the critical genes are conserved only among jawed vertebrates, although adaptive systems have evolved convergently in other lineages [10]. Loss-of-function mutation of some immune genes has been associated with increased susceptibility to fatal virus infection before reproductive maturity [11], arguing strongly that antiviral activity is among their evolved functions [12].

Recent studies have suggested that antiviral immune systems directed by small RNAs are active in mammals (Table 1) [13–18]. Like proteinaceous innate immune receptors, some RNA-based antiviral systems are broadly expressed, such as those directed by microRNAs (miRNAs), whereas others, such as those reportedly mediated by siRNAs, are active in specialized cell types. Some small RNA–directed responses are amplified in a feedforward manner reminiscent of the expansion of virus-specific lymphocyte clones in adaptive immunity. In general, however, the reported small RNA–directed immune mechanisms defy categorization as innate or adaptive; for example, some can respond immediately after viral infection, yet also have high specificity mediated by complementary base pairing with their target sequences. The evolutionary history of the RNAs guiding these systems also run the gamut from tightly constrained mature tRNAs, as ancient as cellular life [19], to piRNAs, notable for the remarkable plasticity of their targeting capacity even within a species [20]. Evidence of small RNA–directed immunity has emerged in the postgenomic era; our understanding of how the constituent components interact at scales larger than individual cells to produce organismal phenotypes is more limited than for classical immunology [21]. Functional and evolutionary categorizations are thus less useful to organize an overview of RNA-guided immunity than they are for protein-guided systems; here, we will summarize the current knowledge regarding antiviral immune functions directed by small noncoding RNAs grouped on the basis of their biogenesis. Notably, biochemical definitions of these molecules and their cofactors preceded recognition of their antiviral properties of the RNAs in each case.

**Table 1:** Classification of antiviral small RNAs.

	Source	Target	RNA vs. Target	Function
**siRNA**	Exogenous viral RNA	Exogenous viral RNA	1 vs 1	Cleavage of viral RNA
**miRNA**	Endogenous mammalian genome	Endogenous host RNA Exogenous viral RNA	Network	Regulation of gene expression (adaptation to antiviral state)
**piRNA**	Endogenous viral elements	Endogenous or exogenous viral RNA	1 vs 1; amplification possible via “ping pong” cycling	Cleavage of viral RNA; transcriptional silencing of integrated viruses
	cDNAs of exogenous viral RNAs			
	Direct processing of viral RNAs			
**tRNA**	Endogenous mammalian genome	Endogenous or exogenous viral RNA	1 vs 1	Multiple proposed mechanisms of action, e.g., steric hindrance of primer-binding site

cDNA, complementary DNA; miRNA, microRNA; piRNA, PIWI-interacting RNA; siRNA, small interfering RNA; tRNA, transfer RNA.

### Viral RNA elimination by siRNA-directed RNA interference

**Fig 1:**
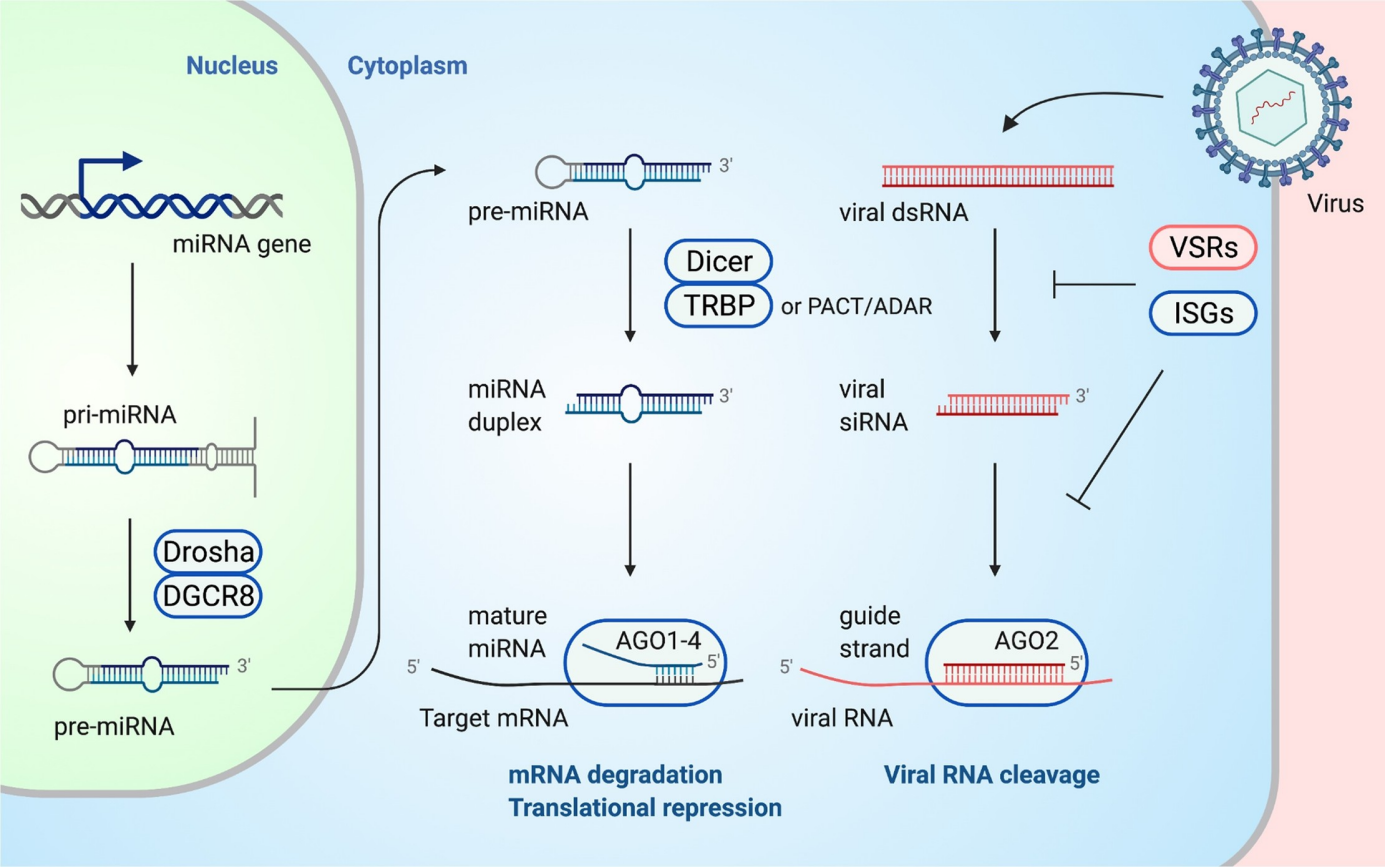
Overview of antiviral siRNA/miRNA pathways and inhibitory factors. Endogenous miRNAs are transcribed from the genome as pri-miRNAs and are processed into pre-miRNAs by the Drosha/DGCR8 complex. Pre-miRNAs are exported to the cytoplasm and are processed into miRNA duplexes by Dicer and its partner protein complex. Viral RNAs are capable of being processed by the same ribonucleases; however, some VSRs or ISGs are reported to inhibit the processing of small RNAs in mammalian cells. Mature miRNAs or siRNAs silence both endogenous and exogenous RNAs loaded on AGO proteins in collaboration with various RNA silencing regulators for the antiviral functions. Endogenous nucleic acids and proteins: blue; viral nucleic acids and proteins: red. AGO, argonaute; dsRNA, double-stranded RNA; ISG, IFN-stimulated gene; miRNA, microRNA; pre-miRNA, precursor miRNA; pri-miRNA, primary transcript; siRNA, small interfering RNA; VSR, viral suppressor of RNAi.

The best studied example of small RNA–directed immunity is that guided by viral siRNAs (Fig 1). When RNA or DNA viruses infect cells, viral origin double-stranded RNAs (dsRNAs) can be generated either as replication intermediates or as structured regions of viral genetic material. These dsRNAs can be cleaved by the endoribonuclease Dicer, which is well conserved in chordates [22], into siRNAs, which are characteristically 21 to 24 nucleotides (nt) in length with a 2-nt 3′ overhang. One of the resulting strands, the guide strand, is loaded onto an argonaute clade protein (AGO), also an endonuclease, and base pairs with complementary viral RNA. AGOs are core components of the RNA-induced silencing complex (RISC) and some trigger the cleavage of the viral RNA, resulting in the repression of viral replication. Here, we refer to this system as antiviral RNAi, and it functions as a potent defense system in plants and invertebrates [23,24]. In contrast, whether antiviral RNAi functions as a meaningful aspect of mammalian defenses against viruses is controversial. Recent reviews have cataloged the evidence in favor of this activity [25], and, since 2020, experiments with knockout mouse challenge have shown that Ago4 is involved in mouse defense against influenza virus [26] and Ago2 with defense against Nodamura virus (NoV) lacking B2 (see below) [27]. The mechanisms proposed to limit the effectiveness of antiviral siRNAs in mammals are potentially relevant to the antiviral activity of other small RNAs as well, so here we will focus on them.

Two types of mechanisms, those enabled by viral suppressors of RNAi (VSRs) and IFN signaling, reportedly limit the effectiveness of viral siRNAs in mammalian immunity. Several viral genes are reported as VSRs. The deletion or mutation of VSRs such as B2 protein of NoV [28] or nonstructural protein 1 (NS1) of influenza A virus (IAV) [29] leads to the production of virus-derived siRNAs (vsiRNAs) in human or mouse somatic cells, whereas infection by the intact viruses does not (Table 2) [28–46]. Several proteins, such as virion protein 35 (VP35) of Ebola or Marburg virus, can prevent vsiRNA accumulation in cells infected by viruses lacking their native VSR, e.g., IAV lacking NS1 [31]. Furthermore, the deletion of these VSRs can repress viral replication, and the repression is recovered by Dicer or AGO2 deficiency. In addition, inhibitory peptides VSRs can induce the production of vsiRNAs [47], suggesting that the VSRs increase viral replication by inhibiting vsiRNA processing [41]. Human Dicer, however, also regulates the maturation of endogenous miRNAs, so Dicer deficiency may be assumed to affect posttranscriptional gene regulation by endogenous miRNAs as well, while AGO2 deficiency has little effect on the overall miRNA population. In other words, even in settings in which human Dicer has an antiviral effect, it is difficult to interpret whether this antiviral effect is derived from viral RNA cleavage by vsiRNAs or the regulation of antiviral gene expression by endogenous miRNAs. VSRs have been shown to act at various steps in antiviral RNAi pathways, including sequestration of dsRNA and inhibition of RNA silencing factors by RNA/protein or protein/protein interaction [30,45,46].

**Table 2:** List of representative VSRs in mammalian cells.

Virus	VSR	Mechanism	Reference
IAV	NS1	dsRNA binding	Li and colleagues [29]
NoV	B2	dsRNA binding	Sullivan and colleagues [28]
HIV-1	Tat	dsRNA binding	Bennasser and colleagues [32]
HBV	HBx	dsRNA binding	Chinnappan and colleagues [33]
HCV	Capsid	Interaction with Dicer	Wang and colleagues [34]
	NS2	dsRNA binding	Zhou and colleagues [35]
SFV	Capsid	dsRNA binding	Qian and colleagues [36]
Ebola virus	VP30, VP35, VP40	Interaction with Dicer, TRBP, or PACT/dsRNA binding	Haasnoot and colleagues [30] Fabozzi and colleagues [37]
Marburg virus	VP35	dsRNA binding	Li and colleagues [31]
Dengue virus	NS4B	dsRNA nonbinding	Kakumani and colleagues [38]
	NS3	Interaction with HSC70	Kakumari and colleagues [39]
	NS2A	dsRNA binding	Qiu and colleagues [40]
HEV71	3A	dsRNA binding	Qiu and colleagues [41]
CoV	N	dsRNA binding	Cui and colleagues [42]
SARS-CoV	7A	-	Karjee and colleagues [43]
SARS-CoV-2	N	-	Mu and colleagues [44]
Adenovirus	VA1 RNA, VA2 RNA	Binding to Dicer	Lu and Cullen [45] Andersson and colleagues [46]

Note that we list only the first report, to our knowledge, that each of these proteins has VSR activity.

CoV, coronavirus; dsRNA, double-stranded RNA; HBV, hepatitis B virus; HCV, hepatitis C virus; HEV71, human enterovirus 71; HIV-1, human immunodeficiency virus 1; IAV, influenza A virus; NoV, Nodamura virus; NS1, nonstructural protein 1; SARS-CoV, severe acute respiratory syndrome–associated coronavirus; SARS-CoV-2, severe acute respiratory syndrome–associated coronavirus-2; SFV, Semliki forest virus; VP30, virion protein 30; VSR, viral suppressor of RNAi.

Antagonism between IFN and RNAi pathways may also limit the effectiveness of the latter. Deletion of either MAVS (also as known as IPS-1), an essential adaptor protein in IFN signaling triggered by RIG-I or MDA5, or IFNAR1, an IFN receptor on the cell surface, enables dsRNA-induced RNAi in MEF [48]. dsRNA-induced RNAi uncovered in MAVS-depleted MEF is again lost upon AGO2 depletion, indicating that IFN signaling masks AGO2-dependent, dsRNA-induced RNAi in mammalian somatic cells [48]. Some IFN-stimulated genes (ISGs) encoded in mammalian genomes might function as endogenous RNAi suppressors, as IFN treatment represses small RNA processing by Dicer in noninfected cells [48,49]. At least one ISG, LGP2, has been shown to repress Dicer activity [49–51]. IFN stimulates hundreds of ISGs, and additional ISGs may also be involved in the suppression of RNAi. While IFN-elicited effects may block vsiRNA production in some settings, antagonism by IFN may not be the only explanation for restricted antiviral RNAi activity in mammals. For example, deletion of RIG-I or MDA5 abrogated IFN production but did not uncover vsiRNA production during HeLa cell infection by positive-sense RNA viruses Sindbis virus (SINV), yellow fever virus 17D vaccine strain (YFV17D), and coxsackievirus B3 (CBV3) [52], arguing that the suppressive activity of ISGs cannot be evoked as the reason vsiRNA are not produced under those conditions. Notably, infection by these viruses did not limit the ability of lentivirus-expressed shRNAs to decrease luciferase expression, arguing that these viruses do not encode a VSR effective under these conditions.

Several observations may harmonize the apparently discordant observations in this field. Whereas in general differentiated cells produce IFN upon viral infection or poly(I:C) treatment, some pluripotent or multipotent stem cells do not [53] and show attenuated response to exogenous IFN [54]. Pluripotent stem cells are able to process viral dsRNA into vsiRNA during EMCV or NoV infection [55], and the vsiRNA production is inhibited by B2 protein of NoV [56]. Mouse embryonic stem cells (mESCs) have an attenuated IFN response but show dsRNA-induced RNAi, whereas this activity is not detected after differentiation [48]. Mouse neural progenitor cells infected with Zika virus (ZIKV) or SINV showed production of vsiRNAs [57,58]. The recent discovery of alternative Dicer isoforms with antiviral activity in mouse and human stem cells extends this concept [59]. Notably, some ISGs are constitutively expressed in pluripotent or multipotent human stem cells, although these show different expression patterns in different tissue’s stem cells [60]. It is possible that some stem cells have antiviral RNAi activity, but others do not. In addition, when interpreting the inhibitory effect of IFN or ISGs for antiviral RNAi, it is important to consider whether global translational repression has been induced by protein kinase R (PKR) under the conditions used. PKR is activated by viral dsRNA and induces global translational repression [61,62], and activation of PKR can occur upstream and potentially independently of IFN signaling via RLRs [63]. It is reported that PKR is constitutively activated in Dicer-deficient mESCs and IFN production is elevated in Dicer-deficient mESCs compared to WT mESCs [59,64,65], suggesting that differential viral replication in Dicer-deficient mESCs results from the combination of viral RNA elimination by antiviral RNAi and PKR-induced translational repression of viral proteins used in viral replication. In the setting of PKR activation, IFN production may not necessarily correspond to high protein expression of ISGs. Intriguingly, several VSRs have also been reported to act as repressors of IFN production and repressors of PKR activation by interfering with its dsRNA-binding activity [29,66]. In summary, accumulation of dsRNA has various consequences that limit cellular permissiveness to virus replication. Interactions between dsRNA-responsive pathways preclude simple statements of the physiological relevance of mammalian vsiRNAs, especially those that would characterize vsiRNAs as standing apart from simultaneously induced pathways long recognized as antiviral, such as IFN.

### Posttranscriptional gene regulation by miRNA-directed RNA silencing

miRNAs direct posttranscriptional gene regulation and, in so doing, often regulate antiviral responses in a manner consistent with this being among their evolved functions (Fig 1). miRNAs are approximately 22-nt endogenous noncoding RNAs [67]. In contrast to the roughly equivalent number of protein-coding genes [68], the human and mouse genomes encode 1,917 and 1,234 miRNA precursors (pre-miRNAs), respectively, which are processed into 2,656 or 1,978 mature miRNAs [69]. miRNAs exhibit expression patterns specific to each tissue [70], suggesting that miRNAs are involved in the complex gene regulatory networks characteristic of each tissue. miRNAs are transcribed from the genome as primary transcripts (pri-miRNAs) and are processed into pre-miRNAs by a Drosha/DGCR8 complex named microprocessor [71]. Pre-miRNAs are exported by Exportin-5/Ran-GTP to the cytoplasm and are processed into miRNA duplexes by Dicer [72–74]. The miRNA biogenesis machinery is sometimes directly involved in antiviral defense; Dicer regulates both vsiRNA and miRNA production in virus-infected cells, and Drosha is reportedly able to cleave pri-miRNA-like structures in viral RNAs [75,76]. miRNAs do not induce RNA cleavage even if incorporated into AGO2 but instead selectively regulate RNAs by destabilizing them or repressing their translation. miRNAs recognize target RNAs by complementary base pairing at the position 2 to 7 or 2 to 8 from the 5′ end, called the seed region [77]. As the seed region is only 6 or 7 nt long, a single miRNA may repress hundreds of genes simultaneously [78].

Some miRNAs target endogenous mRNAs in a manner that promotes antiviral responses, while the others target viral RNAs directly (Table 3) [79–87]. As examples of the former, miR-221/-222 increases after macrophage stimulation by tumor necrosis factor-α (TNF-α) and inhibits human immunodeficiency virus-1 (HIV-1) entry by down-regulating CD4 mRNA [79], and miR-3614, expressed in macrophages, increases after DENV infection and represses infection by targeting ADAR1 mRNA [80]. As an example of the latter, miR-323 increases after IAV infection and represses infection by targeting viral PB1 RNA [85].

**Table 3:** Examples of antiviral immunity directed by endogenous miRNAs.

	Expression	Target	Function	Virus	Cells tested	Reference
**miR-221/-222**	Increased by TNFα	Host CD4	Inhibition of virus entry	HIV-1	Human primary macrophages	Lodge and colleagues [79]
**miR-3614**	Increased by viral infection	Host ADAR1	Reduction of virus infectivity	DENV	Human primary macrophages	Diosa-Taro and colleagues [80]
**miR-183 cluster**	-	Host negative regulators of IRF3 and STAT1	Promotion of IFN production	VSV	HepG2 cells	Singaravelu and colleagues [81]
**miR-340**	Decreased by viral infection	Host RIG-I, OAS2	Reinforcement of antiviral immunity	IAV	A549 cells	Zhao and colleagues [82]
**miR-106b**	Decreased by viral infection	Host initiator and executioner caspases	Enhancement of cell death of virus-infected cells	SeV	HeLa cells	Takahashi and colleagues [83]
**miR-378/-30e**	Decreased by IFN	Host Granzyme B and perforin	Reinforcement of NK cell cytotoxicity	-	Human NK cells	Wang and colleagues [84]
**miR-323**	Increased by IAV infection	Viral PB1	Repression of viral replication	IAV	HEK293T cells, MDCK cells	Song and colleagues [85]
**miR-1/-30/-128/-196-296/-351/-431/-448**	Increased by IFN	Viral RNA genome	Repression of viral replication	HCV	Huh7 cells, murine primary hepatocytes	Jopling and colleagues [86] Pedersen and colleagues [87]
**miR-122**	Decreased by IFN	5′ noncoding region of viral genome				

DENV, dengue virus; HCV, hepatitis C virus; HIV-1, human immunodeficiency virus 1; IAV, influenza A virus; IFN, interferon; miRNA, microRNA; NK, natural killer; SeV, Sendai virus; TNFα, tumor necrosis factor-α; VSV, vesicular stomatitis virus.

As for siRNA, mutual regulation with the IFN system has also been reported for miRNA. IFN stimulation leads to distinct regulation of miRNA pools, with some increased and some decreased [84,87]. For example, IFN-induced miR-1/-30/-128/-196/-296/-351/-431/-448 display antiviral activity against hepatitis C virus (HCV), whereas miR-122, which is essential for HCV replication, is reduced by IFN [86,87]. miR-378 and -30e are repressed by IFN leading to activation of natural killer (NK) cells, enhancing their cytotoxicity [84]. In addition to changing abundance, epi-transcriptional modification of miRNAs can influence their antiviral activities. For example, human cytomegalovirus (HCMV) infection induces the expression of ADAR1 p110, which edits an adenosine in the seed of miR-376a to inosine [88]. This modifies the miRNA’s targeting and influences the susceptibility of infected cells to elimination by NK cells.

Both global regulation of miRNAs and regulation of specific populations of miRNAs may enhance virus elimination or link the innate and adaptive immune systems. Global decrease of miRNAs induces proinflammatory cytokine production [89]. Specific decrease in the group of miRNAs bound by TRBP up-regulates apoptosis regulatory genes and results in increased apoptosis of virus-infected cells [83]. For example, miR-106b targets several caspase mRNAs, including both initiator and executioner caspases, and its decrease causes relative up-regulation of these transcripts and enhances apoptosis of virus-infected cells [83]. miRNAs can also be released from infected cells and transported in extracellular vesicles (EVs), suggesting the possibility that miRNAs function like cytokines to transmit signals and modify gene expression of other cells in both a paracrine and autocrine manner [90]. Indeed, miRNAs have been shown to function as paracrine agonists by activating TLRs to promote IFN secretion [91,92], indicating that miRNA dynamics influence both intracellular and intercellular networks relevant to antiviral states. Like cytokine levels or antibody titers, tracking EV-packaged miRNAs has been proposed as a biomarker for prediction of antiviral efficacy [93]; for example, serum levels of miR-122 in patients infected with HCV reflects the success of IFN/ribavirin therapy [94]. Lastly, some viruses encode miRNAs; these are understood to counteract antiviral immunity and promote virus replication and are outside our scope but have been reviewed elsewhere [95].

### CRISPR-like adaptive immunity directed by piRNAs

**Fig 2:**
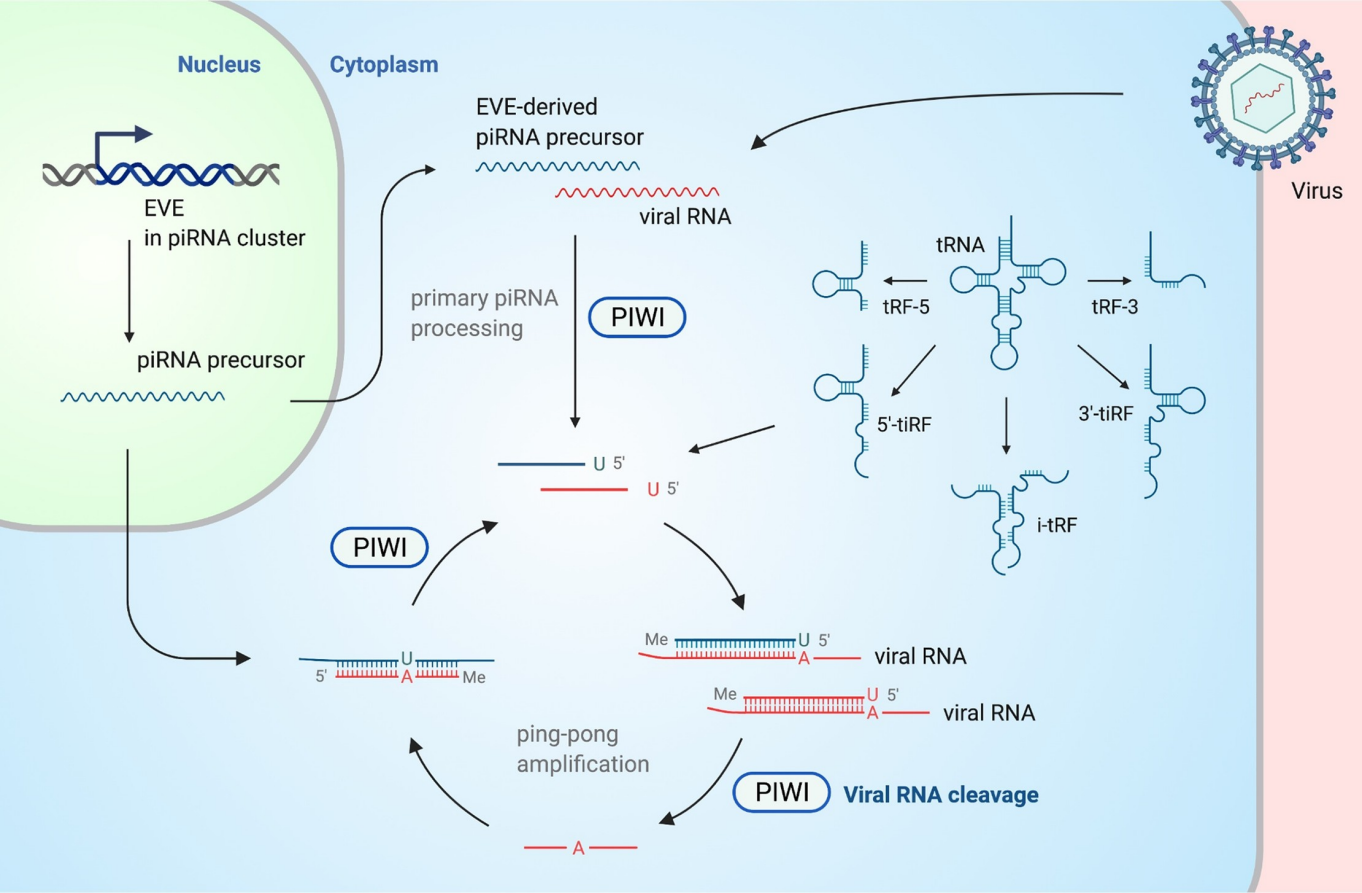
Overview of antiviral piRNA pathway and antiviral tRFs. piRNAs with antiviral potential can be generated via several mechanisms: primary processing of piRNA cluster transcripts that contain an EVE, direct non-piRNA guided cleavage of viral RNA via a Piwi protein (vpiRNA), EVE-derived piRNA- or vpiRNA-guided cleavage of viral RNAs via a ping-pong amplification loop, or PIWI interactions with virus-homologous noncanonical piRNAs such as tRFs. EVE, endogenous viral element; piRNA, PIWI-interacting RNA; tRF, tRNA fragment; vpiRNA, virus-specific PIWI-interacting RNA.

piRNAs are small RNAs that have been experimentally demonstrated to interact with Piwi proteins. piRNAs are often 24 to 31 nt, and most are transcribed from regions known as piRNA clusters. The biogenesis, nucleotide composition, and modification pattern of many piRNAs is distinct from other small RNAs (Fig 2). piRNAs can be generated in at least 2 different ways: phased pathways, in which piRNAs are sequentially produced from the same RNA strand, and ping-pong amplification, in which a pair of piRNAs are produced from dsRNA formed by the hybridization of a piRNA with its target [96]. Mature piRNAs form piRNA-induced silencing complexes (piRISCs) with Piwi proteins, and these complexes can induce heterochromatin formation or directly cleave their target RNA. As piRISC targets are often transcripts of transposable elements (TEs) and defects in the piRNA system often lead to gametogenesis failure, the canonical function of piRNAs is considered to be maintenance of the genome integrity of germ cells. So far, bona fide mammalian piRNAs have been found in the gonads, although Piwi proteins and/or small RNAs sharing features of piRNAs have been associated with functions in some somatic tissues, such as regulation of innate immunity in the lungs [97].

The evolutionary origins and replication strategies of some viruses and TEs overlap; viruses and TEs can be considered together as mobile genetic elements, and recent reports in nonmammalian organisms suggest that both can be repressed by the piRNA system [98,99]. Sources for antiviral piRNAs include direct processing of exogenous viral RNAs [100], RNA fragments (e.g., tRNA; see below), or transcripts from endogenous viral elements (EVEs), including those within piRNA clusters. About 9% of a human’s genome is accounted for by EVEs, which are virus-derived sequences having undergone horizontal gene transfer (HGT) into the germline of our ancestors. EVEs, such as endogenous retroviruses (ERVs), may give rise to antiviral piRNAs. Koala retrovirus-A (KoRV-A) is found as both an infectious virus and as ERV provirus copies in some Koala germline genomes. Similarly, ecotropic murine leukemia viruses (MuLVs) recently entered some laboratory mouse germlines as ERVs and, in some cases, still encode particles capable of infecting mouse cells. Sense-stranded 24 to 31 nt testes-expressed small RNAs are derived from unspliced KoRV-A transcripts and may suppress this virus in the gonad, and sense-biased piRNAs made from ecotropic MuLV are similarly detected in the testes of mice whose genomes contain such proviruses [18]. Not all EVEs are derived from retroviruses; endogenous bornavirus-like nucleoprotein elements (EBLNs) are derived from an ancient relative of bornaviruses, which are negative-strand RNA viruses [101]. EBLNs entered rodent and primate lineages independently, yet EBLN-derived piRNAs are detected in gonads of both mouse and marmoset. This is remarkable given both the limited number of EBLNs and the small fraction of the genome that encodes piRNAs and likely indicates that EBLN integrations into piRNA clusters have been selectively retained [17]. Production of antiviral piRNAs from EVEs is reminiscent of an adaptive immune strategy in prokaryotes, the clustered regularly interspaced short palindromic repeat (CRISPR)/CRISPR-associated (Cas) system [102]. In both cases, sequences from “non-self” mobile elements are incorporated into the genome by HGT to function in immune memory [103].

In many somatic or cancer cells, epigenetic manipulations that result in ERV expression also induce IFN signaling in an RLR-dependent manner [104,105]. In piRNA-deficient germ cells, ERV and other TE up-regulation is associated with meiotic arrest and apoptosis [106], though the precise signals triggering these defects remain unclear. Mouse germ cells express TLR3 and MDA5 and produce IFN and up-regulate ISGs upon poly(I:C) stimulation [107,108]. IFN overproduction in transgenic mice leads to male-specific sterility and increased apoptosis of spermatocytes [109]. An intact piRNA pathway may be necessary to prevent ERVs, inevitably expressed during the epigenetic erasures inherent to germ cell development, from triggering innate immune pathways incompatible with germ cell survival. Notably, in neural progenitor cells, TE expression upon DNA demethylation is met with a dramatic up-regulation of piRNA biogenesis genes [110], suggesting that some somatic cells may remain poised to generate piRNAs upon perturbation.

### tRNAs and tRNA fragments (tRFs) as antiviral molecules

tRNAs regulate virus infections and TEs in addition to, or connected with, their canonical role in protein synthesis. Viruses generally do not encode tRNAs, while in humans, tRNAs are among the most abundant RNAs [111]. There are 10 times as many tRNA genes as codon–anticodon pairs, and considerable diversity exists in tRNA sequences besides the anticodon loop. Most tRNA genes cluster in discrete loci, the largest of which is adjacent to the major histocompatibility complex class I (MHC-I) coding region on chromosome 6 and encodes nearly all major tRNAs [112]. tRNAs are 70 to 90 nt in length and can be processed into tRFs (usually ranging from 14 to 32 nt) by ribonucleases including angiogenin and Dicer in a stimulus-dependent manner [111,113–115]. tRFs derive from various points along the tRNA cloverleaf structure; 5′ and 3′ end-derived tRF halves (tRF-5 and tRF-3, respectively) have most often been reported to regulate viral infection.

Endogenous and exogenous retroviruses, as well as the TEs derived from them, use tRNAs as primers to initiate reverse transcription. IAP is an active mouse TE in the family of lysine tRNA-primed ERVs, and at least one IAP locus encodes functional membrane fusion machinery and can produce infectious particles [116]. tRFs were shown to inhibit IAP, as well as other TEs dependent on reverse transcription (MusD/ETn), by two mechanisms: posttranscriptional silencing and blocking interaction with tRNAs necessary for priming reverse transcription [117]. tRFs may indirectly influence replication of non-retroviruses; respiratory syncytial virus (RSV) infection increases tRF production in infected cells, and while one of the resulting tRFs can increase virus replication, the net effect of these changes on RSV infection outcome in cells remains unknown [118]. A tRF derived from the pre-tRNA-Ser 3′ trailer sequesters host RNA chaperone La/SSB and represses La/SSB-dependent viral gene expression during HCV infection [119]. While these reports suggest specific tRFs’ involvement in virus infection, global changes to tRNAs and tRFs are also linked to antiviral immunity; protecting tRNAs from stress-induced cleavage by angiogenin in T cells was recently shown to be an activity of schlafen 2, deletion of which results in greater susceptibility to viral infection [120]. This activity is reminiscent of the anti-HIV activity of schlafen 11, which is also dependent on regulation of the tRNA pool [121].

Some tRFs are reported to be loaded into Ago or Piwi proteins. tRF-5s and tRF-3s associate with all Ago paralogues in humans as tRNA-derived miRNAs (td-miRNA) [122]. Several have additionally been shown to guide sequence-specific silencing of cellular [123] and viral [124] transcripts. tRF halves serve as a major source of tRNA-derived piRNA (td-piRNA) in silkworm [125], but the biogenesis of td-piRNAs in mammals remains to be fully clarified. Gtsf1, the murine homologue of drosophila Asterix, is a piRNA biogenesis factor that preferentially binds tRNA over piRNA and may suppress tRNA-primed retroelements [126], although the mechanism remains incompletely defined. Several tRFs are reported to bind Piwil4 (Hiwi2) in both normal and transformed human cells [127]. PIWIL2 (HILI) has been reported to interact with intact tRNAs and induce translational repression of HIV-1 via this activity [128], though HILI may also interact with tRFs that were undetected using the methods applied [129].

### Current and future applications of small RNAs as nucleic acid medicine

Small RNAs have favorable characteristics as antiviral therapeutics. Various types of 18 to 30 nt length RNAs have been approved by the FDA as nucleic acid medicines as of April 2021: 9 antisense oligonucleotides (ASOs), 1 aptamer, 4 siRNAs, and 1 CpG oligonucleotide. The specific sequences and modifications of these nucleic acid medicines are not present in nature, but each harnesses an endogenous system directed by small RNAs. As powerfully demonstrated in development of mRNA vaccines against COVID-19, which took only 45 days from the publication of the SARS-CoV-2 draft genome sequence to shipment of a product for clinical trials, nucleic acid medicines can be chemically synthesized in large amounts quickly. Target and off-target recognition of small RNAs can be rationally designed and screened in silico against databases of nucleic acids present in uninfected human cells. This stands as an advantage over existing platforms for designing protein-based therapeutics, notably monoclonal antibodies, despite advances in structure prediction and molecular dynamic simulations. Among the approved small RNA medicines, ASO and siRNA were rationally designed based on predicted complementarity to the target RNA, whereas the sequence of the approved aptamer was experimentally screened using SELEX [130]. Among the small RNAs reviewed in previous sections, only siRNAs have been approved as medicines; miRNA, piRNA, and tRNA-based medicines remain a therapeutic frontier. Delivery of nucleic acid medicines to their target tissues was a major early hurdle; small RNAs are quickly degraded in the serum by RNases or subject to glomerular filtration. However, recent advances in drug delivery systems such as lipid nanoparticles or nucleic acid glycosylation have addressed these challenges, as mRNA vaccination has again demonstrated. Understanding how small RNAs, such as EV-packaged miRNAs, act as paracrine regulators in physiological mammalian immunity holds promise for additional improvements. The personalizability of nucleic acids is another promising feature for individualized medicine. For example, a personalized ASO, Milasen, was designed and used for a patient with Batten disease. This required less than a year from sequencing the patient’s genome, which enabled rational design, to symptom improvement [131]. Similar to human genome sequencing, which is transitioning to routine clinical use, virus sequencing infrastructure will substantially increase as a consequence of the current pandemic, so designing small RNAs based on the viral genotypes circulating in an individual or population is becoming more realistic. Similar to those in development for cancer, small RNAs targeting known pathogenic mutations might be used to anticipate and limit viral evolution [132]. Harnessing the piRNA pathway for antiviral therapy would be complicated by the limited expression of Piwi proteins in somatic cells, yet the possibility of “looped” production between the delivered nucleic acid medicine and a target viral quasispecies is also notable with respect to evolution of resistance. Compared to delivery of Cas9 ribonucleoproteins, which is now clinically applicable [133], delivery of human Piwi ribonucleoproteins would carry the theoretical advantage of immune tolerance to the endonuclease component and could potentially be coupled to “endogenous-inspired” delivery systems [134].

## Conclusions

Uncovering the mechanisms that enable human immunity to viruses has led to many success stories in development of antiviral therapies, from plant-derived compounds to IFN therapy to CCR5 antagonists to monoclonal antibodies. Clarifying the role of small RNA–directed antiviral immune systems in mammals will likely provide additional successes; the recently described antiviral actions of miRNAs, piRNAs, and tRNAs are especially promising. In parallel to advances in the basic science, mRNA-based therapies and prophylactics have seen accelerated development since the SARS-CoV-2 pandemic, seemingly overcoming previous barriers to nucleic acid medicines. Small RNAs have big potential for the future therapeutic applications.
